# KASP Genotyping as a Molecular Tool for Diagnosis of Cassava-Colonizing *Bemisia tabaci*

**DOI:** 10.3390/insects11050305

**Published:** 2020-05-14

**Authors:** Everlyne N. Wosula, Wenbo Chen, Massoud Amour, Zhangjun Fei, James P. Legg

**Affiliations:** 1 International Institute of Tropical Agriculture, P.O. Box 34441 Dar es Salaa, Tanzania; a.massoud@cgiar.org (M.A.); j.legg@cgiar.org (J.P.L.); 2 Boyce Thompson Institute, 533 Tower Rd, Ithaca, NY 14853, USA; chenwenbo1020@gmail.com (W.C.); zf25@cornell.edu (Z.F.); 3 USDA-ARS Robert W. Holley Center for Agriculture and Health, 533 Tower Rd, Ithaca, NY 14853, USA

**Keywords:** Kompetitive Allele-Specific PCR, NextRAD, SNPs, whitefly, cassava

## Abstract

*Bemisia tabaci* is a cryptic species complex that requires the use of molecular tools for identification. The most widely used approach for achieving this is the partial sequencing of the mitochondrial DNA cytochrome oxidase I gene (*COI*). A more reliable single nucleotide polymorphism (SNP)-based genotyping approach, using Nextera restriction-site-associated DNA (NextRAD) sequencing, has demonstrated the existence of six major haplogroups of *B. tabaci* on cassava in Africa. However, NextRAD sequencing is costly and time-consuming. We, therefore, developed a cheaper and more rapid diagnostic using the Kompetitive Allele-Specific PCR (KASP) approach. Seven sets of primers were designed to distinguish the six *B. tabaci* haplogroups based on the NextRAD data. Out of the 152 whitefly samples that were tested using these primer sets, 151 (99.3%) produced genotyping results consistent with NextRAD. The KASP assay was designed using NextRAD data on whiteflies from cassava in 18 countries across sub-Saharan Africa. This assay can, therefore, be routinely used to rapidly diagnose cassava *B. tabaci* by laboratories that are researching or monitoring this pest in Africa. This is the first study to develop an SNP-based assay to distinguish *B. tabaci* whiteflies on cassava in Africa, and the first application of the KASP technique for insect identification.

## 1. Introduction

The whitefly, *Bemisia tabaci* (Gennadius; Hemiptera: Aleyrodidae), with a host range of over 1000 plant species, is considered one of the most damaging crop pests worldwide. The greatest damage caused is by the vectoring of over 300 plant viruses [1]. In Africa, *B. tabaci* transmits viruses that cause cassava mosaic disease (CMD) and cassava brown streak disease (CBSD) [2,3]. The combined damage resulting from infection with these two diseases is estimated to cause cassava yield losses amounting to 50% in East and Central Africa, causing annual losses equivalent to more than US$1 billion [4]. 

*Bemisia tabaci* is genetically complex [5], with many distinct genetic groups that have been identified based on sequences of the mitochondrial cytochrome oxidase I (*COI*) gene [6,7]. The occurrence of biotypes or host races of the whitefly *B. tabaci* was described in the 1950s after the discovery that morphologically indistinguishable populations of *B. tabaci* differed with respect to host range, host–plant adaptability, and plant virus transmission capabilities [8]. At that time, identification of *B. tabaci* relied on morphological characterization involving the examination of slide-mounted specimens of fourth instar nymphs [9]. However, some characteristics of the fourth instar are influenced by prevailing environmental conditions encountered during the crawler stage and leaf morphology [10,11]. This led to confusion in the nomenclature, identification, and classification of *Bemisia*, and ultimately resulted in many putative species of *Bemisia* being synonymised under the single species name, *Bemisia tabaci* [11]. 

The inconsistency in relying on morphological characteristics to classify *Bemisia* led to the exploration of new molecular techniques to characterise *B. tabaci* groups, such as the use of esterase isozyme polymorphisms [8], as well as a variety of DNA markers, including RAPDs, PCR–RFLPs and AFLPs [12]. Advancement in molecular tools led to the application of sequencing of 16S rRNA, cytochrome oxidase I (*COI*) gene portions in the mitochondrial genome, and the nuclear ribosomal intergenic spacer 1 (*ITS1*), a non-coding sequence, for genetic characterization of whiteflies [5,6,12,13]. Microsatellite markers have also been developed to study population structure, revealing broad geographic affiliations and levels of substructure [14]. 

The *COI* marker has become the most commonly used marker for phylogenetic studies of *B. tabaci* [5,6,12,13], and it was used to propose that the *B. tabaci* complex is composed of at least 24 distinct species [6]. However, *COI* has the disadvantage that it is a single locus that is maternally inherited; therefore, it is unlikely to produce an adequate genetic resolution to differentiate populations, and it does not provide a full delineation of phylogenetic history [15]. In some scenarios, mitochondrial barcoding has been found to overestimate the number of species or scale of divergence where there are nuclear mitochondrial DNA pseudogenes (NUMTs) [16,17]. In addition, data from 2184 nuclear orthologs obtained from whole-genome sequencing of whiteflies representing the major clades of *B. tabaci* revealed the existence of fewer putative species as opposed to the much larger number reported with *COI* [18]. These recent findings cast doubt on the reliability of *COI* for delimiting *B. tabaci* cryptic species [17,18].

Several studies have reported that single nucleotide polymorphisms (SNPs) are informative and affordable genetic markers [19,20,21]. Low cost and simple methods for de novo SNP discovery in model and non-model species have increased rapidly due to the availability of reduced representation genome sequencing techniques [22]. One of these approaches is a recent genotyping technology, Nextera restriction-site-associated DNA (NextRAD) sequencing, which allows for the generation of sequence data from small organisms [23]. This approach has been successfully utilized to study fine-scale population genetics and diversity in insects [24,25,26,27].

Interest in cassava colonizing *B. tabaci* in Africa was occasioned by the severe outbreak of cassava mosaic disease (CMD) in Uganda in the 1990s [28]. Previously, two strains of *B. tabaci* were reported in West Africa, the cassava-associated strain designated “cassava” and a strain associated with other crops [29]. In Uganda, two distinct strains of *B. tabaci* were reported based on esterase profiles—those found on cassava and those on other crops such as sweet potato and cotton [30]. Host-association studies later suggested that the cassava strain was more-or-less restricted to cassava, as previously observed in the Ivory Coast [29], while the sweet potato/cotton strain was polyphagous on other crops but could not colonize cassava [31]. The cassava strain was later found to have two distinct genotypes based on *COI* sequencing that were designated Ug1 and Ug2, with the latter shown to be associated with the severe CMD epidemics of the 1990s [32]. Further studies on cassava *B. tabaci* from sub-Saharan Africa (SSA) reported the existence of five genetically distinct groups (SSA1–5) based on *COI* sequencing [33]. SSA1 was further divided into five subgroups, based on phylogenetic analyses of *COI* [34,35]. Most recently, an analysis of >63,000 genome-wide single nucleotide polymorphisms (SNPs) obtained from cassava-colonizing *B. tabaci* from Africa revealed the existence of six haplogroups, designated as SSA2, SSA4, SSA-CA, SSA-ESA, SSA-WA and SSA-ECA [24,25]. These SNP genotyping studies [24,25] represent the most comprehensive assessment made to date of the genetic diversity of this cryptic species complex. 

The high cost and time required for NextRAD sequencing means it cannot be used for routine monitoring of cassava-colonizing *B. tabaci* in Africa. There is, therefore, a need to develop an alternative rapid, accurate and affordable SNP-based tool for whitefly identification. In this study, we developed a diagnostic Kompetitive Allele-Specific PCR (KASP) assay to distinguish the six haplogroups of cassava *B. tabaci*. KASP is a technique that was developed by LGC Genomics Teddington-UK, and it is based on allele-specific oligo extension and fluorescent resonance energy transfer (FRET) for signal generation. The assay uses allele-specific fluorescent labelled primers that allow for end-point fluorescent reads enabling the bi-allelic scoring of single nucleotide polymorphisms at specific loci. (https://biosearch-cdn.azureedge.net/assetsv6/KASP-genotyping-chemistry-User-guide.pdf). KASP assays are low cost, high-throughput and have high specificity and sensitivity relative to other markers [36]. 

The objective of the study described here was to develop SNP markers to distinguish the six major haplogroups of cassava *B. tabaci* whiteflies using a KASP PCR technique and to validate these markers by comparing them with genotyping by NextRAD sequence data.

## 2. Materials and Methods 

### 2.1. Whitefly NextRAD Sequences

We previously collected 95 cassava-colonizing whitefly samples from eight African countries (Burundi, Cameroon, Central African Republic (CAR), Democratic Republic of Congo (DRC), Madagascar, Nigeria, Rwanda and Tanzania) [24]. An additional 190 adult whitefly specimens were collected from cassava fields between 2015 and 2018 from 10 additional countries (Benin, Ghana, Kenya, Liberia, Malawi, Mozambique, Sierra Leone, Togo, Uganda and Zambia) and four countries (Cameroon, DRC, Nigeria and Tanzania) that either had few samples in our previous study or harboured high whitefly diversity. Genomic DNA of the 190 whiteflies was used to construct NextRAD libraries by SNPsaurus, LLC (http://snpsaurus.com/) [23]. Raw reads from NextRAD sequencing (190 samples combined with 95 samples) [24] were first processed to remove adaptor and low-quality sequences. The cleaned reads were then aligned to the SSA-ECA genome, and only uniquely mapped reads were used for SNP calling using TASSEL5 [37]. The final filtered SNPs (63,770) were obtained from a total 243 *B. tabaci* cassava whiteflies that produced quality sequences out of the combined 285 samples. 

Downstream analyses revealed the existence of six major cassava *B. tabaci* haplogroups designated as sub-Saharan Africa East and Central Africa (SSA-ECA; comprising samples identified as SSA1-SG1, SSA1-SG2 and SSA1-SG1/SG2 based on *COI*), sub-Saharan Africa East and Southern Africa (SSA-ESA; samples identified as SSA1-SG3 based on *CO*I), sub-Saharan Africa Central Africa (SSA-CA; samples identified as SSA1-SG1 based on *COI*), sub-Saharan Africa West Africa (SSA-WA; samples identified as SSA1-SG1 and SSA1-SG5 based on *COI*), sub-Saharan Africa 2 (SSA2; samples identified as SSA2 and SSA3 based on *COI*) and sub-Saharan Africa 4 (SSA4; samples identified as SSA4 and SSA3 based on *COI*) [24,25]. The SNPs were visualised in Microsoft Excel, and a total of 217 whiteflies (SSA-ECA = 60; SSA-WA = 52; SSA2 = 33; SSA4 = 10; SSA-ESA = 50; SSA-CA = 12) were manually searched for homozygous alleles that distinguished the six haplogroups. 

### 2.2. Primer Design

SNPs to distinguish the six newly designated cassava-colonising *B. tabaci* haplogroups (SSA-ECA, SSA-ESA, SSA-WA, SSA-CA, SSA2 and SSA4) were identified (Table 1), and genome portions of ~2000 bp containing the SNPs were extracted from the *Bemisia tabaci* isolate SSA1 (SSA-ECA) whole-genome assembly (GenBank accession PGTP01000000). Seven sets of primers were designed (BTS99-319, BTS22-762, BTS55-473, BTS141, BTS613, BTS46-203 and BTS1161) (Table 2) and optimised to amplify genome fragments (390 to 1150 bp) containing the target SNPs to distinguish the six haplogroups. Genome sequence portions of ~200 bp containing target SNPs were sent to LGC Genomics-UK to design the KASP primers (Table 3). PCR products generated using conventional primer sets were then used as the DNA template to test and optimise the KASP primers (Table 3). The KASP technique was tested for effectiveness to distinguish the six newly designated cassava-colonizing *B. tabaci* haplogroups (SSA-ECA, SSA-ESA, SSA-WA, SSA-CA, SSA2 and SSA4). A total of 152 whitefly samples from Nigeria, Ghana, Benin, Sierra Leone, Liberia, Cameroon, Malawi, Mozambique, Kenya, Tanzania, Uganda and the Democratic Republic of Congo (DRC) were tested using KASP. These were selected from the same DNA extracts that were used for SNP genotyping (NextRAD) [25] with the aim of comparing KASP with NextRAD. Conventional PCR products of the six primers were generated and subsequently used as templates in KASP genotyping. 

### 2.3. Conventional PCR and KASP Assay

The conventional PCR reaction mixture (10 µL) contained 1 µL template DNA, 5 µL OneTaq Quick-Load 2X Master Mix with Standard Buffer, 0.24 µL of primer (0.25 mM), 0.4 µL MgCl_2_ (25 mM) solution, and 3.36 µL of sterile water. A total of 35 cycles of amplification were carried out, and conditions were the same for all set of primers: denaturation at 95 °C for 5 min and 94 °C for 40 s, annealing temperature at 58 °C for 30 s, and extension at 72 °C for 45 s, and a final extension at 72 °C for 10 min and held at 10 °C.

The KASP reaction mixture (10 µL) contained 5 µL 2X KASP master mix, 0.14 µL KASP primer assay mix and 5 µL DNA template (1 µL of PCR product/DNA extract + 4 µL of sterile water). KASP genotyping was performed in a Strategene MX 3000P (Agilent Technologies, California-USA). The following cycling conditions were used: Stage 1: 30 °C 60 s (pre-read); Stage 2: 94 °C for 15 min hot-start Taq activation (1 cycle); Stage 3: 94 °C for 20 s, 61 °C (61 °C decreasing 0.6 °C per cycle to achieve a final annealing/extension temperature of 55 °C) for 60 s (10 cycles); Stage 4: 94 °C for 20 s, 55 °C for 60 s (29 cycles); Stage 5: 94 °C for 20 s, 57 °C for 60 s (3 cycles); Stage 6: 37 °C for 60 s (1 cycle, cooling) followed by an end-point fluorescent read. These conditions were used for five primers (BTS99-319, BTS22-762, BTS55-473, BTS141, BTS1161), while Stage 3: 94 °C for 20 s, 68 °C (68 °C decreasing 0.6 °C per cycle to achieve a final annealing/extension temperature of 62 °C) was used for two primers, BTS613 and BTS46-203. 

The quality of genotyping cluster plots was visually assessed, and only samples in distinct clusters were considered for manual SNP calling, using the MxPro software incorporated in the Strategene MX 3000P unit and KlusterCaller (LGC Genomics, Teddington, UK). Genotyping profiles obtained from KASP assays were compared to genotype data from NextRAD to ensure only matching genotypes were considered. 

## 3. Results

**Figure 1:**
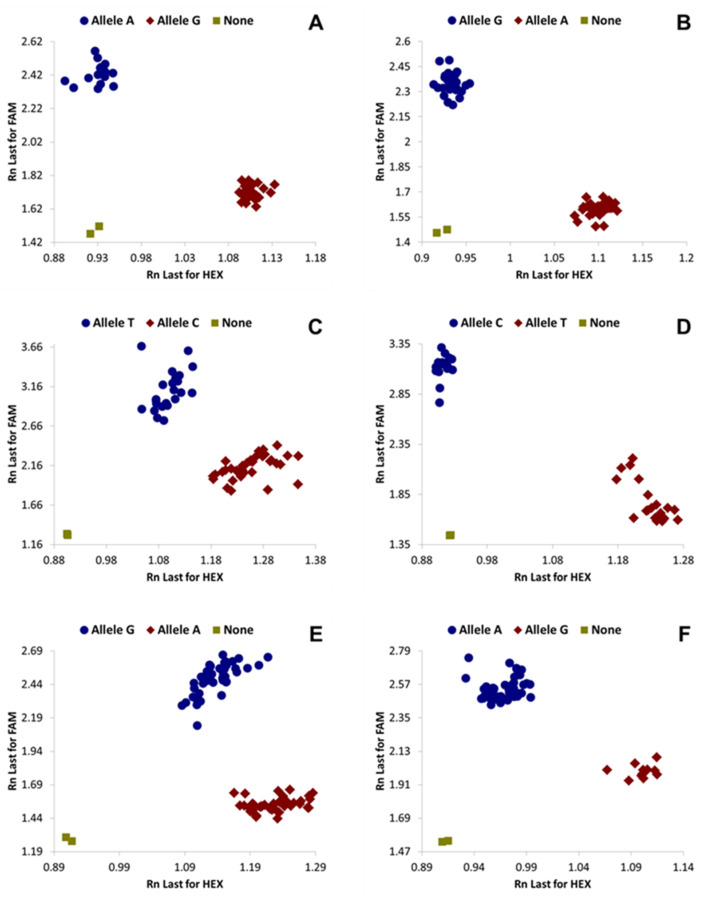
Cluster plots of six KASP SNP primers distinguishing cassava *Bemisia tabaci* whiteflies. Blue and red represent the two distinct alleles, while olive green represents no template negative controls. (**A**) = BTS99-319 (SSA-ECA and SSA-WA vs. SSA-ESA, SSA-CA, SSA2, SSA4); (**B**) = BTS22-762 (SSA-ECA vs. SSA-WA); (**C**) = BTS141 (SSA2 and SSA4 vs. SSA-ECA, SSA-WA, SSA-ESA, SSA-CA); (**D**) = BTS55-473 (SSA2 vs. SSA4, SSA-ECA, SSA-WA, SSA-ESA, SSA-CA); (**E**) = BTS613 (SSA-ESA and SSA-CA vs. SSA-ECA, SSA-WA, SSA2, SSA4); (**F**) = BTS46-203 (SSA-ESA vs. SSA-CA).

**Figure 2:**
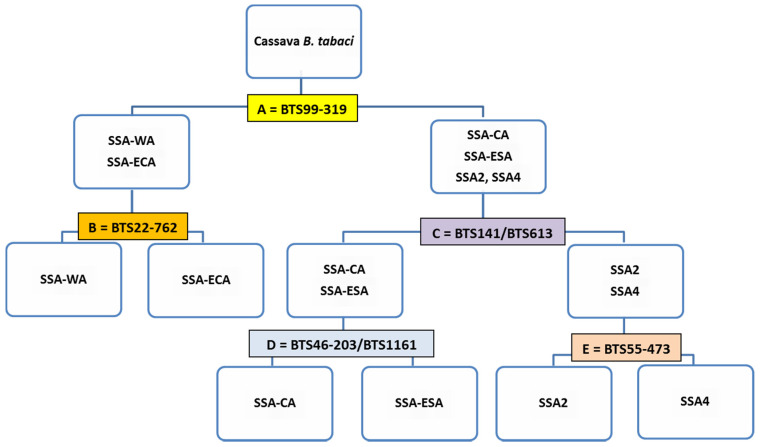
KASP diagnostic assay flow chart for designating cassava *Bemisia tabaci* whiteflies into six major haplogroups (A = primer that separates SSA-ECA and SSA-WA from the other four haplogroups; B = primer that separates SSA-ECA from SSA-WA; C = primers that separate SSA2 and SSA4 from SSA-ESA and SSA-CA; D = primers that separate SSA-ESA from SSA-CA; E = primer that separates SSA2 from SSA4 and also SSA2 from the other five haplogroups.

The KASP genotyping results were consistent with NextRAD genotyping (99.3%) with the exception of one sample. The SNP genotyping clusters for the selected six primers for representative samples are presented (Figure 1). The conventional primers BTS99-319F/R amplified a ~940-bp fragment with a clear single bright band for cassava whiteflies but did not amplify any of the non-cassava *Bemisia tabaci* (MED and IO) and *B. afer* that were tested in our laboratory. This primer is therefore used to first split African cassava *B. tabaci* from other species in addition to providing PCR templates for subsequent KASP assays. The KASP diagnostic flow chart (Figure 2) shows the steps to be followed to split cassava whiteflies into the six populations.

*Separating SSA-ECA and SSA-WA from all other cassava* B. tabaci *haplogroups*. The SNP primers BTS99-319 used in KASP gave genotyping results for the 152 samples that were consistent with NextRAD haplogroups and that corresponded with NextRAD alleles in 151 (99.3%) of the samples. The SSA-ECA and SSA-WA haplogroups (73 samples) clustered as A:A and those in the other four haplogroups (SSA-ESA, SSA-CA, SSA2 and SSA4) (78 samples) clustered as G:G. Only one sample from Malawi—designated as SSA-ESA with NextRAD alleles (G:G)—was designated as A:A by KASP genotyping.

*Splitting SSA-ECA and SSA-WA*. The conventional primers BTS22-762F/R amplified a fragment of ~880 bp. KASP genotyping with these primers was consistent with NextRAD for all 41 (100%) samples that were designated as SSA-WA (G:G) and 31 out of 32 (97%) samples that were designated as SSA-ECA (A:A). A single sample from Kenya designated as SSA-ECA by NextRAD was identified as SSA-WA by KASP. These primers were designed to separate SSA-ECA from SSA-WA; the other four haplogroups have mixed alleles at this SNP position, as confirmed by both NextRAD and KASP results. The samples in the SSA-ECA group were from DRC, Kenya, Tanzania and Uganda, while those in SSA-WA were from Benin, Ghana, Liberia, Nigeria and Sierra Leone.

*Distinguishing SSA2 and SSA4 from other cassava* B. tabaci *haplogroups.* The conventional primers BTS141F/R amplified a ~1150-bp fragment. Genotyping of the 152 samples with KASP primers gave consistent results with NextRAD in 151 (99.3%) cases. KASP identified 34 samples as SSA2 or SSA4 (T:T) and 117 as other haplogroups (C:C). A single mismatched sample from DRC typed as SSA4 by NextRAD was heterozygous by KASP. The 117 samples belonging to the other four haplogroups had consistent results for both genotyping methods. Two samples grouped as SSA2 by NextRAD and typed in the same way with KASP had missing NextRAD alleles.

*Splitting SSA2 and SSA4*. Conventional primers BTS55-473F/R amplified a ~815-bp fragment. KASP genotyping of the 152 samples was 100% consistent with NextRAD. Twenty-six SSA2 samples (T:T) were separated from SSA4 (8 samples) and also from the rest of the groups. These primers can be used to directly separate SSA2 from the other five haplogroups. The samples in the SSA2 group were from Cameroon, DRC, Ghana, Kenya and Sierra Leone, while those in the SSA4 group were from Cameroon and DRC.

*Distinguishing SSA-ESA and SSA-CA from other cassava* B. tabaci *haplogroups.* The conventional primers BTS613F/R amplified a ~730-bp fragment. KASP genotyping of the 152 samples was 100% consistent with NextRAD. There were 45 samples in the SSA-ESA and SSA-CA group (G:G) and 107 samples in the group combining the other four haplogroups (A:A). 

*Distinguishing SSA-ESA from SSA-CA.* Conventional primers BTS46-203F/R amplified a ~390-bp fragment. These primers were tested only to separate SSA-ESA from SSA-CA and were not used with the other four haplogroups. KASP genotyping of 45 of these samples identified 39 as SSA-ESA and two as SSA-CA, which is consistent with NextRAD. The remaining four samples, two each from SSA-ESA and SSA-CA based on NextRAD grouping and typed homozygous by NextRAD alleles, were shown to be heterozygous when using KASP. These heterozygous samples were tested by an alternative primer set BTS1161 that also separates SSA-ESA from SSA-CA, and the samples were consistent with NextRAD as either SSA-ESA or SSA-CA. Amplification results using this primer for SSA-ESA samples from East Africa and some from Southern Africa were consistent with NextRAD, but 8 samples (4 from Malawi and 4 from Mozambique) out of 41 were designated as heterozygous and one sample from Mozambique was identified as SSA-CA although NextRAD identified it as SSA-ESA. SSA-ESA samples originated from Kenya, Malawi, Mozambique and Tanzania, whilst SSA-CA samples were from eastern DRC and western Tanzania. 

Summarised results show that overall there was a very high level of concordance between NextRAD and KASP diagnoses (Table 4 and Table 5). Detailed information on the country, *CO*I identity, NextRAD grouping and KASP result is provided for all 152 samples in Table S1. 

## 4. Discussion

*Bemisia tabaci* is a cryptic species complex with diverse members that have different biological properties. Accurate identification of these species is critical for the effective management of whiteflies, both as pests and as virus vectors. Most recently, an analysis of >63,000 genome-wide single nucleotide polymorphisms (SNPs) obtained from cassava-colonizing *B. tabaci* from Africa revealed the existence of six haplogroups, designated as SSA2, SSA4, SSA-CA, SSA-ESA, SSA-WA and SSA-ECA [24,25]. These data have now been used to design a simple KASP diagnostic assay to distinguish the six haplogroups. The method allows for the identification of these haplogroups in laboratory procedures lasting a matter of hours and with no requirement for sequencing. As such, we suggest that the method is appropriate for adoption across sub-Saharan Africa, where routine identification of cassava *B. tabaci* whiteflies is required. The SSA-ECA haplogroup is of particular interest as it is predominant in regions currently affected by the severe CMD and CBSD pandemics [25] and commonly occurs in super-abundant populations.

KASP results were consistent with NextRAD (99.3%), with only one sample out of the 152 not matching. A comparison with *COI* reveals that 18.4% (28 out of 152) of the samples were misidentified by *COI*. The SSA1-SG1 samples were predominantly placed in SSA-ECA, but 8.5% (13 out of 152) of these were actually SSA-WA. This indicates that authors still using *COI* will often be lumping two distinct haplogroups (SSA-ECA and SSA-WA) together as SSA1-SG1. Other samples that were misidentified include 7 SSA-ECA that were designated as SSA1-SG2, 3 SSA2 as SSA3, 2 SSA-CA as SSA1-SG1, 1 SSA-WA as SSA2, 1 SSA2 as SSA4 and 1 SSA-ECA as SSA1-SG1/SG2. This high misidentification rate of samples using *COI* indicates that it is unreliable for identifying the major genetic groups of cassava *B. tabaci* whiteflies, as has been highlighted in previous studies [24,25]. The KASP assay had 99.3% consistency with NextRAD and is more accurate and reliable compared to *COI* sequencing. The single sample (0.7%) that was scored heterozygous by KASP but homozygous by NextRAD is an expected infrequent occurrence because allelic dropout can occur during RAD sequencing and it increases for low read loci [38,39]. 

The whitefly samples directly used in KASP assays were from 12 countries in all major cassava-growing regions in Africa, indicating that this tool will be reliable in identifying cassava *B. tabaci* whiteflies from all of the major cassava-growing regions of the continent. The current protocol is designed to include conventional PCR to generate the DNA template for KASP with conventional primers. This step is necessary for whiteflies because individual insects produce low amounts of DNA that may not be of sufficiently good quality for the KASP assay. Attempts to use directly-extracted DNA as a template for KASP produced inconsistent results with some samples producing distinct clusters while others failed. Using a DNA template from a specific region with distinguishing SNPs can also eliminate the incidence of unspecific primer binding to non-target genome regions. This KASP assay set up did not present any challenges that required optimisation, and samples were resolved into the expected clusters just by following through the provided LGC Genomics protocol for all the SNP primers that were tested. 

The availability of the cassava whitefly reference genome [25] made it straightforward to design this assay as it was possible to locate the SNPs and the >50-bp flanking genome regions on both sides of the SNP as recommended by LGC Genomics. In other systems, the lack of a reference genome can make assay design significantly harder [40]. Test results for the six SNP primers recommended yielded a total of 721 tests, out of which 714 (99%) matched the NextRAD genotyping. This level of accuracy is comparable to a study in which 8 SNPs tested on 10 samples of Eurasian beavers yielded 76 out of 80 (95%) samples that matched RAD sequencing results [40]. Our whitefly study described here is the first to use KASP genotyping on an insect. This also means that it should provide a valuable baseline against which to measure the merits of subsequent applications of KASP to identify genetic groupings of insects. This KASP assay will be used for the routine characterization of whiteflies collected from cassava in Africa, which will be expected to cluster into the six populations. Since it is anticipated that there may be distinct populations of cassava *B. tabaci* occurring at a low frequency that have not currently been sampled, additional NextRAD sequencing will be required at occasional intervals to allow for updates to be made to the KASP diagnostic in order to ensure that it remains as accurate and comprehensive as possible.

## 5. Conclusions

This study presents a KASP assay for the routine monitoring of cassava *Bemisia tabaci* in sub-Saharan Africa. Since this assay has demonstrated results consistent with NextRAD sequencing, it can provide a rapid and reliable analysis of cassava *B. tabaci* that will allow for same-day in-house genotyping in local laboratories that have limited access to sequencing technologies. 

The KASP assay developed here has important practical developmental applications. Since there remains a concern that the haplogroup SSA-ECA may pose a risk for the continued spread of severe CMD and CBSD pandemics, the KASP diagnostic described could be an important component within early warning systems to track the spread of potentially dangerous *B. tabaci* populations. Finally, the KASP diagnostic represents an important application of comprehensive genomics data for *Bemisia* on cassava in Africa. As other datasets become available for *Bemisia* populations elsewhere in the world, there are likely to be similar opportunities to develop and apply KASP diagnostics more widely for *Bemisia* identification and monitoring as part of pest and vectored virus disease management programmes.

## Figures and Tables

**Table 1:** Single nucleotide polymorphisms (SNPs) for distinguishing six haplogroups of cassava-colonising *Bemisia tabaci* whiteflies.

KASP Primers	Position in Genome	SSA-ECA	SSA-WA	SSA-ESA	SSA-CA	SSA2	SSA4
BTS99-319	Intergenic region	A:A	A:A	G:G	G:G	G:G	G:G
BTS22-762	Ssa12858; exon	A:A	G:G	-	-	-	-
BTS141	Intergenic region	C:C	C:C	C:C	C:C	T:T	T:T
BTS55-473	Intergenic region	C:C	C:C	C:C	C:C	T:T	C:C
BTS613	Intergenic region	A:A	A:A	G:G	G:G	A:A	A:A
BTS46-203	Intron of Ssa00724	-	-	A:A	G:G	-	-
BTS1161	Intron of Ssa00849	-	-	C:C	A:A	-	-

**Table 2:** Conventional PCR primers for amplifying genome portions containing target SNPs for cassava *Bemisia tabaci* whiteflies.

Primer	Sequence	Band (bp)	Accession	Range
BTS99-319F	TTTCTGGAGGTATGATGTT	940	PGTP01000606.1	849,024–849,984
BTS99-319R	GTTGGCTTGTTTTTCTTTG			
BTS22-762F	CAAACGAACACAACCGCAA	880	PGTP01001647.1	62,324–63,244
BTS22-762R	CAGGGACGTACACAAAATAA			
BTS141F	TCCTCAGCAGTGTCTTTT	1150	PGTP01000392.1	896,170–897,370
BTS141R	TCTACGTTGTGTTGTCGG			
BTS55-473F	ACCCCACCAAATATCTCAC	815	PGTP01143321.1	559,651–560,491
BTS55-473R	GGCATTCCAGCAAAATATACA			
BTS613F	CATTCCGCTTTCCATCCTC	730	PGTP01000317.1	28,760–29,515
BTS613R	CCTTCTCTTGTCGAACAT			
BTS46-203F	CGAGGGCTAAAGAATAATAC	390	PGTP01000379.1	1,505,366–1,505,786
BTS46-203R	TTCAGAACGAATGAGAAGG			
BTS1161F	TTATTTTCGGTGGTGCGTC	500	PGTP01001427.1	371,644–372,644
BTS1161R	GATGATGAGGGTAGAGTT			

**Table 3:** Kompetitive Allele-Specific PCR (KASP) primers distinguishing six haplogroups of cassava-colonizing *Bemisia tabaci* whiteflies.

ID	Primer AlleleX	Primer AlleleY	Primer Common	Size bp
BTS99-319	CTCAAATTTAAAATACGATTTCAATTACCATT	CTCAAATTTAAAATACGATTTCAATTACCATC	GCTGCATATTATACCGCATGAAAGCTAAA	40
BTS22-762	CAGTCAATTAAAAGACGTCTCGCTAA	CAGTCAATTAAAAGACGTCTCGCTAG	GTCGCTGTCTTGTTTTCCCTCCAT	50
BTS141	TACTATTTCTAGCAAAGCGAATTTAAATCATA	CTATTTCTAGCAAAGCGAATTTAAATCATG	GGAGTGCTATAAAGCGACCTATATGTAT	36
BTS55-473	CCGCACAGGAGACCCAAGTC	ACCGCACAGGAGACCCAAGTT	TAATAAGCCCGACATGCCGCTCTTT	38
BTS613	GGTAGAGCGGCGCTTGGTC	ATGGTAGAGCGGCGCTTGGTT	ACTTCGGCTTTGAACTTCCCGCAAA	30
BTS46-203	GGTGCATCGTATCGCATCTCTGA	GTGCATCGTATCGCATCTCTGG	CATATAACTACGCGCAACGCAACGTA	62
BTS1161	AAGTCTTGCTGCTATGGCTTAGTTC	AAAGTCTTGCTGCTATGGCTTAGTTA	CCCCATGTAGAGCTCCAGGTAAAAT	60

**Table 4:** KASP diagnosis results summary for cassava *Bemisia tabaci* whiteflies.

KASP Primers	NextRAD Identity	KASP Diagnosis	Accuracy	Mismatch
(i) BTS99-319	A:A (SSA-ECA/SSA-WA) (73)	A:A (74)	99.3%	0.7% (Malawi)
	G:G (SSA-ESA/SSA-CA/SSA2/SSA4) (79)	G:G (78)		
(ii) BTS22-762	G:G (SSA-WA) (41)	G:G (42)	98.6%	1.4% (Kenya)
	A:A (SSA-ECA) (32)	A:A (31)		
(iii) BTS141	T:T (SSA2/SSA4) (35)	T:T (34) C:T (1)	99.3%	0.7% (DRC)
	C:C (SSA-ECA/SSA-WA/SSA-CA/SSA-ESA) (117)	C:C (117)		
(iv) BTS55-473	T:T (SSA2) (26)	T:T (26)	100%	0%
	C:C (SSA4) (8)	C:C (8)	100%	0%
(v) BTS613	G:G/G:A (SSA-ESA/SSA-CA) (45)	G:G (45)	100%	0%
	A:A (SSA-ECA/SSA-WA/SSA2/SSA4) (107)	A:A (107)	100%	0%
(vi) BTS46-203	A:A (SSA-ESA) (41)	A:A (39) A:G (2)	95.1%	4.9% (Kenya, Tanzania)
	G:G (SSA-CA) (4)	G:G (2) A:G (2)	50%	50% (DRC)
(vii) BTS1161	A:A (SSA-ESA) (41)	C:C (33) C:A (8)	80.5%	19.5% (Malawi, Mozambique)
	G:G (SSA-CA) (4)	A:A (4)	100%	0%

**Table 5:** Summary of cassava *Bemisia tabaci* whiteflies identified by KASP vs. NextRAD.

		NextRAD Identity					
		SSA-ECA	SSA-WA	SSA-CA	SSA-ESA	SSA2	SSA4
KASP Diagnosis	SSA-ECA	31 ^a^					
	SSA-WA	1 ^b^	41 ^a^				
	SSA-CA			4 ^a^			
	SSA-ESA				41 ^a^		
	SSA2					26 ^a^	
	SSA4						8 ^a^

^a^ Number of samples matching for KASP vs. NextRAD; ^b^ Number of samples mismatching for KASP vs. NextRAD

## Supplementary Materials

The following are available online at https://www.mdpi.com/2075-4450/11/5/305/s1, Table S1: NextRAD vs. COI vs. KASP genotypying of Cassava *Bemisia tabaci* whiteflies.
